# Optimization of input parameters to a CN neuron model to simulate its activity during and between epileptic absence seizures

**DOI:** 10.1186/1471-2202-16-S1-P280

**Published:** 2015-12-18

**Authors:** Parimala Alva, Lieke Kros, Oscar H.J Eelkman Rooda, Chris I De Zeeuw, Rod Adams, Neil Davey, Freek E Hoebeek, Volker Steuber

**Affiliations:** 1 Science and Technology Research Institute, University of Hertfordshire, Hatfield AL10 9AB, UK; 2 Department of Neuroscience, Erasmus Medical Center, Rotterdam, the Netherlands; 3 Netherlands Institute for Neuroscience, Royal Dutch Academy for Arts and Sciences, Amsterdam, the Netherlands

## 

**Figure 1:**
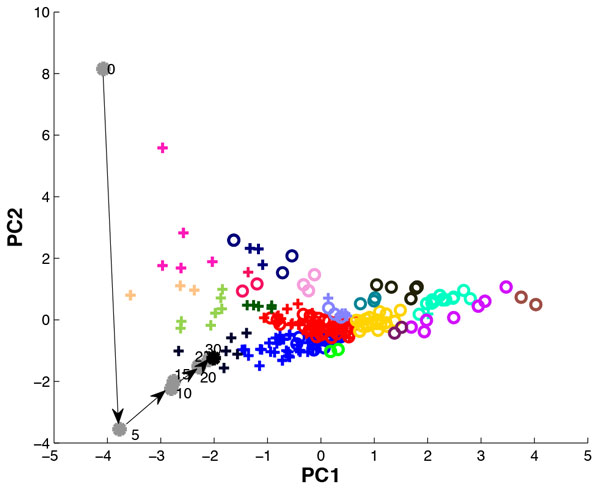
**2D projection, using principal component analysis, of the clusters formed as a result of GNG clustering of the CN neuron's interictal activity using CV, burst-like spike ratio and mode ISI**. (+) indicate cells that participate in the seizure and (o) indicate the cells that do not participate based on the measures, FFT based Z-score and modulation frequency. The black (+) indicate the selected "participating" cluster for the EA based optimization. The grey dots indicate the output data points for every 5th generation of the EA and the arrows show the progression of the output data points from the initial position indicated by 0 to the interictal cluster center.

Absence seizures are often attributed to the synchronized oscillatory activity in the thalamo-cortical regions of the brain, and they can be detected by the presence of spike-wave-discharges (SWDs) in the electroencephalogram (EEG). The cerebellar nuclei (CN), which have afferent connections to these regions, may also play a role in the propagation of these seizures. Some CN neurons in *Cacna1a*^tottering ^(tg) mice phase-lock their spiking activity to the peaks of the SWDs in the EEG during the absence seizures. These CN neurons are deemed to "participate" in the absence seizures. To investigate if certain types of CN neuron are more likely to participate, we performed Growing Neural Gas (GNG) clustering [1], using different subsets of the feature set that consisted of CV, mean Cv2, log CV, log-interval entropy, permutation entropy, firing rate, burst index, pause ratio, burst-like spike ratio, mean ISI, mode ISI, median ISI, min ISI of the interictal parts of the spike-trains. The subset of the feature set that produced the best separation of clusters was CV, burst-like spike ratio, and mode ISI (Figure 1).

We then used a morphologically realistic conductance-based model of an excitatory CN projection neuron [2] to simulate the interictal activity of a participating CN neuron. We selected a cluster where all neurons participated in the seizures, indicated by black crosses (+) in Fig. 1, and applied an Evolutionary algorithm (EA) to optimize excitatory and inhibitory input parameters to the CN neuron model such as spike rates, noise, burst parameters, synchronicity, synaptic weights so that the output data point of the EA moved closer to the centre of the selected cluster. The results of the EA indicated that the CN neurons that participated in absence seizures received either a synchronous, bursting inhibitory input or a synchronous, bursting excitatory input. Next, we modified the EA such that the initial input parameters of the CN neuron model resulted in an output data point nearest to the center of the selected interictal cluster, and ran the optimization to move the output data point to the center of the cluster that was formed from the ictal counter-parts of the CN neurons of the selected participating cluster. Surprisingly, a very small change in input parameters could result in a shift from the interictal to the ictal cluster centre and result in a transition to CN neuron activity as observed during seizures. However, when we blocked the Purkinje cell input to the CN neuron model by maintaining the inhibitory synaptic weight at zero, the output data point never reached the center of the ictal cluster. This suggests that blocking Purkinje cell input to the CN neuron can prevent the CN neuron from participating in the absence seizure.
